# *BRCA1/2* Mutation Detection in the Tumor Tissue from Selected Polish Patients with Breast Cancer Using Next Generation Sequencing

**DOI:** 10.3390/genes12040519

**Published:** 2021-04-02

**Authors:** Ewelina Szczerba, Katarzyna Kamińska, Tomasz Mierzwa, Marcin Misiek, Janusz Kowalewski, Marzena Anna Lewandowska

**Affiliations:** 1 The F. Lukaszczyk Oncology Center, Molecular Oncology and Genetics Department, Innovative Medical Forum, 85-796 Bydgoszcz, Poland; nalejskae@co.bydgoszcz.pl (E.S.); kaminskak@co.bydgoszcz.pl (K.K.); 2 Department of Thoracic Surgery and Tumors, Ludwik Rydygier Collegium Medicum in Bydgoszcz, Nicolaus Copernicus University, 85-067 Torun, Poland; kowalewskij@co.bydgoszcz.pl; 3 The F. Lukaszczyk Oncology Center, Department of Prevention and Health Promotion, 85-796 Bydgoszcz, Poland; mierzwat@co.bydgoszcz.pl; 4 Świętokrzyskie Cancer Center, Clinical Department of Gynaecological Oncology, 25-734 Kielce, Poland; mmisiek@me.com

**Keywords:** *BRCA 1/2* genes, hereditary breast cancer, TNBC patients, next-generation sequencing, bioinformatic NGS software, somatic BRCA variant

## Abstract

(1) Background: Although, in the mutated *BRCA* detected in the Polish population of patients with breast cancer, there is a large percentage of recurrent pathogenic variants, an increasing need for the assessment of rare *BRCA1/2* variants using NGS can be observed. (2) Methods: We studied 75 selected patients with breast cancer (negative for the presence of 5 mutations tested in the Polish population in the prophylactic National Cancer Control Program). DNA extracted from the cancer tissue of these patients was used to prepare a library and to sequence all coding regions of the *BRCA1/2* genes. (3) Results: We detected nine pathogenic variants in 8 out of 75 selected patients (10.7%). We identified one somatic and eight germline variants. We also used different bioinformatic NGS software programs to analyze NGS FASTQ files and established that tertiary analysis performed with different tools was more likely to give the same outcome if we analyzed files received from secondary analysis using the same method. (4) Conclusions: Our study emphasizes (i) the importance of an NGS validation process with a bioinformatic procedure included; (ii) the importance of screening both somatic and germline pathogenic variants; (iii) the urgent need to identify additional susceptible genes in order to explain the high percentage of non-*BRCA*-related hereditary cases of breast cancer.

## 1. Introduction

Breast cancer is the most common cancer in women worldwide [1]. Approximately 5–10% of breast cancers are hereditary. Women carrying *BRCA* mutations have an increased risk of breast cancer and/or ovarian cancer development, with a probability of 45–75% and 18–40%, respectively [2,3,4,5].

Hereditary ovarian/breast cancer (HOBC) is frequently caused by founder mutations in the *BRCA1* and *BRCA2* genes. Founder mutations were historically present with high frequencies in small, geographically or culturally isolated groups and are derived from one or more ancestors [6]. A founder effect can be observed in a population characterized by lower genetic diversity, which might be caused by the parental population suffering a dramatic decrease or bottleneck. The parental population could give rise to a larger population in which new variants could occur spontaneously or be transferred from other populations [7].

Due to the high incidence of breast cancer worldwide and its relatively high mortality and morbidity, it is important to implement appropriate screening tests which enable rapid and efficient mutation screening in the *BRCA1* and *BRCA2* genes. Based on the available wide range of technologies, and owing to the presence of the founder effect or a frequent mutation in the screened population, there are two possible approaches to mutation screening.

The first is cascade testing, widely accepted as the most efficient and cost-effective method of point mutation screening that is adequate in a genetically homogenous population [8]. There are many homogenous populations, such Ashkenazi Jews in Israel, for whom a founder mutation effect can be observed (*BRCA1* c.68_69delAG, *BRCA1* c.5266dupC and *BRCA2* c.5946delT) [9], Ireland (*BRCA1* c.427G>T) [10], Iceland (*BRCA2* c.771_775delTCAAA) [11], the South African population (*BRCA1* c.1374delC, *BRCA1* c.2641G>T and *BRCA2* c.7934delG) [12,13] and Holland (Dutch) (*BRCA1* c.2804_2805delAA, *BRCA1* c.4186-1643_4358-985del (common name IVS12-1643del3835) and *BRCA2* c.5579insA) [14]. A mechanism which inactivates *BRCA1* differently from point mutations is Alu-mediated large genomic deletions [15,16]. The most widely known examples of such CNV founder mutations are the *BRCA1* 3.8-kb deletion of exon 13 and the 510-bp deletion of exon 22 present in the Dutch population [15], or a *BRCA1* exon 3–16 deletion, which represents a Danish founder mutation [17]. *BRCA1/2* genomic rearrangements were further investigated in other Caucasian subpopulations: Dutch (27–36%) [15,18], Italian (19%) [19], Danish (9.2%) [17], Czech (6%) [20], Polish (3.1 [21]–3.7% [22]) and French-speaking Canadian (no large genomic rearrangement (LGR) detected) [23].

Over the years, a broad range of PCR-based mutation methods have been used for screening—starting from the historical SSCP [24,25] and protein truncated test (PTT) [26], followed by ASO-PCR and RT-PCR of *BRCA1* mRNA [27], which helped to discover large genomic rearrangements, and subsequently followed by MLPA and CGH [28].

The second approach to *BRCA1* and *BRCA2* mutation testing is the sequencing of all coding regions using next-generation sequencing (NGS). The main advantage of this method is its potential to detect not only founder mutations, but also other ones, including both frequent and rare pathogenic changes. In addition, *BRCA1* and *BRCA2* tests expanded with CNV analysis are available on the market (Entrogen BRCA complete ver. 2 (EntroGen, Inc, Los Angeles, CA, USA), Devyser BRCA (Devyser, Stockholm, Sweden), SureMASTR BRCA Screen (Agilent, Santa Clara, CA, USA)—enhancing the use of NGS in diagnostics. Studies by Gorski et al. (2000) and Perkowska et al. (2003) in the Polish population showed that the *BRCA1* founder effect exists, with a predominant presence of c.181T>G (p.Cys61Gly) and c.5266dupC, although the mutation spectrum is more dispersed [29,30]. Full *BRCA* gene mutation analysis in Polish high-risk families was first postulated in 2003, and NGS studies in this group followed in 2015. Kluska et al., in their study of Genetic Counseling Unit patients with early-onset or familial breast/ovarian cancer, selected 512 cases negative for 11 *BRCA1* and 9 *BRCA2* mutations. *BRCA1/2* testing using NGS technology showed that 52 out of 512 (10%) Polish patients had additional *BRCA1/2* pathogenic variants [31]. There were 367 patients representing only familial breast cancer, among whom 26 patients had additional *BRCA1/2* pathogenic variants detected using NGS in the group studied by Kluska et al. [31]. In a parallel study, 335 Polish patients with triple-negative breast cancer (negative for 3 *BRCA1* mutations) were tested with NGS. The study revealed the presence of deleterious variants in 33 of 335 patients (9.9%) [32]. Finally, Kowalik et al. detected 40 (8.8%) pathogenic variants in a subpopulation of 454 healthy individuals and patients with breast and/or ovarian cancer referred to the Genetic Counseling Outpatient Clinic [33]. Since that time, the first FDA approval for targeted therapy with PARP inhibitors for patients with *BRCA-*positive ovarian cancer has introduced renewed hope, especially for triple-negative or metastatic breast cancer patients, as many inhibitors are under evaluation for their potential clinical benefits against patients with *BRCA*-positive breast cancer. In the present study, we attempted to determine the prevalence of pathogenic or likely pathogenic variants of *BRCA1* or *BRCA2* among the selected group of patients with breast cancer (not diagnosed as mutation carriers with the standard screening procedure in 2003–2015).

## 2. Materials and Methods

### 2.1. Materials

The studied population, comprising patients enrolled in the study between 2003 and 2015, were screened for the presence of known mutations in the *BRCA1* gene (c.5266dupC, c.181T>G, c.4035delA, c.68_69delAG, c.3700_3704delGTAAA) by the National Cancer Control Program supervised by the Department of Health Promotion and Prevention (Franciszek Lukaszczyk Oncology Center, Bydgoszcz, Poland). Patients, negative for mutations in the *BRCA1* gene during the presented targeted testing, who developed breast cancer (between 2003 and 2015) were referred after providing their informed consent.

A total of 75 breast cancer tissue samples (archived between 2003 and 2017) were selected by the Department of Tumor Pathology and Pathomorphology. The study was approved by the Bioethics Committee of the Nicolaus Copernicus University in Torun (KB 844/2018).

### 2.2. DNA Isolation

The percentage of tumor cells in material qualified by the pathologist ranged from 5% to 80%. DNA was isolated from breast cancer tissue fixed in formalin and embedded in paraffin (FFPE) using the QIAamp DNA FFPE Tissue Kit (Qiagen, Hilden, Germany), according to the manufacturer’s protocol. The initial concentration and quality of DNA were measured using NanoDrop1000 (Thermo Scientific, Waltham, MA, USA).

### 2.3. DNA Quality Assessment

The quality and quantity of DNA were evaluated using real-time PCR with Fragmentation Quantification Assay (FQA) CE-IVD (EntroGen) or/and by fluorometric methods using Quantus (Promega) with QuantiFluor^®^ dsDNA System (Promega).

FQA allows the amplification of 37-, 150- and 300-base pair (bp) fragments of isolated double-stranded DNA in a reference location on chromosome 5 and the evaluation of DNA degradation through assessment of DNA concentration (ng/µL), amplifiable copy number for the three amplicon sizes, as well as fragmentation ratio (F ratio). The F ratios 150 bp/37 bp and 300 bp/37 bp provided information on the amount of dsDNA needed as a template for library preparation. Based on the manufacturer’s protocol, we found that archive FFPE material (from 2010–2017) was significantly degraded, but the lack of other biological material forced us to use DNA with an F ratio of <0.5, below the manufacturer’s recommendations.

### 2.4. Library Preparation for Next-Generation Sequencing

Libraries for NGS were prepared using the BRCA Complete CE-IVD test (EntroGen), which allowed the sequencing of all exons of the *BRCA1* and *BRCA2* genes according to the manufacturer’s protocol. The test can detect single-nucleotide variants (SNVs), small insertions and deletions. The BRCA Complete test uses the target enrichment method in order to amplify the desired DNA fragments in 4 separate reactions, which are combined into one. The target enrichment step allows 115 amplicons with an approximate length of 254 bp to be obtained, and it is followed by DNA end preparation and ligation of adaptor sequences to the amplified DNA fragments. All prepared libraries were dual-indexed. The libraries were quantified using the real-time PCR Library Quantification Assay CE-IVD (EntroGen). All libraries were pooled, denatured and diluted to 5 pM together with the control library PhiX. Sequencing was performed on a MiniSeq platform (Illumina) using the MiniSeq Mid Output Kit, 2 × 150 cycles, to obtain reads for both strands.

### 2.5. Bioinformatic Analysis

FASTQ files were generated on the BaseSpace Onsite system. Our goal was to establish an NGS workflow for the automated and reliable assessment of many samples simultaneously for diagnostic purposes. We wanted to evaluate whether the final identification of pathogenic and likely pathogenic variants using different customized bioinformatic programs and platforms would be the same. We used two separate algorithms for the secondary analysis: (1) we analyzed FASTQ files using an automated Illumina TruSeq Amplicon Workflow (v.1.1.0), which utilizes the Isis analysis software (version 2.4.60.20), SAMtools (version 0.1.18), Isis Smith–Waterman (aligner) and Somatic Variant Caller (version 3.2.3). The Isis Smith–Waterman alignment algorithm is based on the length of the amplicon (maximum indel size is 25 bp) and performs local sequence alignments against amplicon sequences specified in the BRCA Complete CE-IVD manifest file (EntroGen, Inc., Los Angeles, CA, USA). Sequences with more than 3 indels are filtered from alignment results. The obtained vcf files including all detected variants were evaluated using Variant Studio 3.0 application (Illumina) and EntroGen Variant Analysis 1.1 Software (EVA) (EntroGen, Inc., Los Angeles, CA, USA); (2) in parallel, we uploaded the FASTQ files onto the OncoKDM platform (OncoDNA). Bioinformatic analysis of the FASTQ files was performed using the OncoKDM aligner and Freebayes variant caller (v1.1.0-3-g961e5f3) on the OncoKDM platform, provided by OncoDNA SA; Belgium Moncodaneum project. Finally, an additional tertiary analysis for selected samples with detected pathogenic variants was performed using the NGeneAnalySys software (Seoul, Korea), which independently re-annotated samples [29]. All variants are described with the following reference sequences: NM_007300.3 (*BRCA1*), NM_000059.3 (*BRCA2*). There was one exception, c.5266dupC, which is the common name of a frequent *BRCA1* variant with reference sequence NM_007294.4; NM_007194.4 (*CHEK2*); NM_024675.4 (*PALB2*).

### 2.6. Sanger Sequencing

All pathogenic variants were evaluated in non-cancerous tissue (FFPE with 0% content of tumor cells) or in blood. Sanger sequencing was carried out using an Applied Biosystems SeqStudio Genetic Analyzer (Thermofisher Scientific, Stockholm, Sweden).

### 2.7. Quality Control Parameters

Overall, 74/75 sequenced samples had uniformity of coverage above 71.5 (mean 80.8%); one sample had 62.3%. Uniformity of coverage for Illumina platforms is described as the percentage of targeted base positions in which the read depth is greater than 0.2 times the mean region target coverage depth. Mean base quality for samples assessed with oncoKDM platform was 68.8. In addition, 13.3% samples randomly selected from 75 analyzed probes were checked with an additional application to control the quality of FASTQ files and alignment using FastQC, Flagstat and samtools stats.

### 2.8. Statistical Analysis

Mann–Whitney U test for statistical analysis was performed for the study purposes.

## 3. Results

### 3.1. Patient Characteristics

The median age was 43 years at the time of diagnosis (75 patients with breast cancer enrolled in the study were negative for the 5 most common *BRCA1* mutations). Pathogenic variants were detected predominantly in patients diagnosed with breast cancer who were under 46 years of age (*n* = 8), and this prevalence observed in the studied subpopulation of 25–69 years of age was statistically significant (Mann–Whitney U test, α = 0.05) (Table 1). Based on the histopathological results, we categorized patients with breast cancer into the following groups: (i) triple-negative patients (ER-, PR-, Her2-; TNBC), (ii) (ER+, PR+, HER2+), (iii) (ER+, PR+, HER2-), (iv) (ER-, PR-, HER2+), (v) other subtypes. As expected, the highest frequency of pathogenic variants of *BRCA1/2* was detected in the TNBC patients (23%) (Table 2). Among the examined patients, the two most frequent breast cancer subtypes were: ER+, PR+, HER2- (*n* = 42) and TNBC (*n* = 15).

A pathologist qualified the FFPE material, which had a varied percentage of tumor cells, ranging from 5% to 80%. Eight detected pathogenic variants were present in samples with a cancer cell content below 41%. However, the majority of the detected pathogenic variants (8/9) were germline, and tumor cell content was not crucial at the moment of detection in the studied population.

### 3.2. Characteristics of Pathogenic Variants

**Figure 1:**
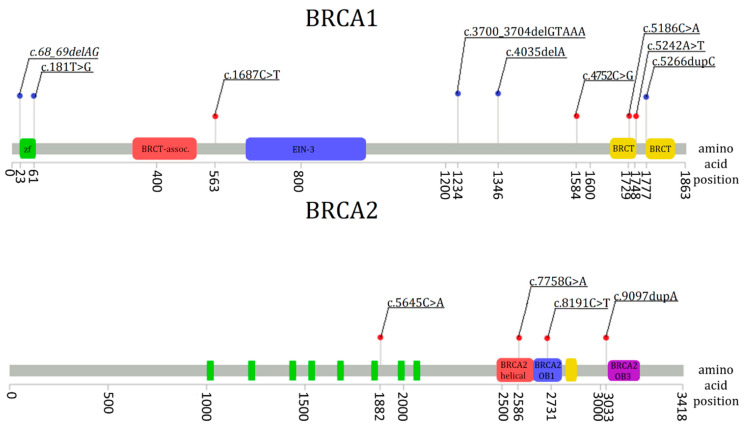
5 *BRCA1* recurrent mutations in the Polish population (blue dots) and pathogenic variants detected in the *BRCA1* and *BRCA2* genes (red dots). Five known mutations in *BRCA1*: c. 68_69delAG, c.181T>G, c.3700_3704delGTAAA, c.4035delA, c.5266dupC. Nine pathogenic variants detected in the study group: c.1687C>T (*BRCA1*), c. 4752C>G (*BRCA1*), c.5186C>A (*BRCA1*), c.5242A>T (*BRCA1*), c.5645C>A (*BRCA2*), c.7758G>A (*BRCA2*), c.8191C>T (*BRCA2*) and c.9097dupA (*BRCA2*). BRCA1 domains: zf—zinc finger C3-HC4; CRCT-assoc.—serine-rich domain associated with BRCT; EIN-3—ethylene-insensitive 3; BRCT—BRCA1 C terminus domain. BRCA2 domains: BRCA2-helical; BRCA2-OB1—oligonucleotide/oligosaccharide binding domain 1; BRCA2-OB3—oligonucleotide/oligosaccharide-binding domain 3.

We detected nine pathogenic variants in the *BRCA1* and *BRCA2* genes in 8 out of 75 patients (10.66%). Among them, eight were missense and one was a frameshift variant (Table 3). All detected pathogenic variants were present in the coding regions (Figure 1) in *BRCA1* exons 10, 16, 18, 19 and in *BRCA2* exons 11, 16, 18, 23.

The pathogenic variant c.4752C>G (*BRCA1*) was found in two unrelated patients. The following detected variants—c.1687C>T (*BRCA1*), c.4752C>G (*BRCA1*), c.5186C>A (*BRCA1*), c.5242A>T (*BRCA1*), c.5645C>A (*BRCA2*), c.7758G>A (*BRCA2*), c.8191C>T (*BRCA2*) and c.9097dupA (*BRCA2*)—had been previously described in the ClinVar database.

In the TNBC patients, we identified the pathogenic variants c.4752C>G (*BRCA1*) in two cases and c.1687C>T (*BRCA1*) in one case. The frequency of the detected pathogenic variants was 66.6%, 79% and 47%, respectively.

A 100% concordance in the detection of pathogenic variants was observed using three different analysis tools (Variant Studio 3.0—Illumina, EVA 1.1—EntroGen, USA, OncoKDM—OncoDNA, Belgium) (100% concordance).

All 75 samples were analyzed using two different algorithms: (1) TruSeq Amplicon Application (Illumina) with somatic variant caller along with EntroGen manifest file; and (2) OncoKDM (OncoDNA SA, Moncodaneum project) with Freebayes variant caller (v1.1.0-3-g961e5f3).

In algorithm (1), the classification of variants was fully consistent, independently of the application used for VCF file annotation (Illumina Variant Studio 3.0 and EntroGen EVA1.1: results consistent for all 75 samples; and NGeneAnalySys: results consistent for 8 samples with pathogenic variants detected). When the results obtained using algorithms (1) and (2) were compared, detections of pathogenic variants were consistent in 98.7% (two variants were not detected by the OncoKDM platform during testing, but subsequently, OncoDNA improved its database, and since October 2019, there has been a 100% correlation).

## 4. Discussion

We detected nine pathogenic variants in 8 (10.66%) out of 75 selected breast cancer patients from families with hereditary breast and ovarian cancer syndrome, whose genetic tests for five *BRCA1* mutations (c.5266dupC, c.181T>G, c.4035delA, c.68_69delAG, c.3700_3704delGTAAA) were negative. It is challenging to compare the frequency of mutations detected in selected groups because of the different study inclusion criteria (e.g., discrepancy between preliminary assessments of pathogenic variants that exclude patients from the study).

According to Kluska et al., pathogenic and likely pathogenic germline *BRCA1/2* variants were detected in the blood in 7.1% of 367 patients with familial breast cancer in the Polish population [31]. Although the percentage of pathogenic and likely pathogenic variants detected was slightly lower, the authors performed a broader preliminary assessment and genotyped 11 known pathogenic variants in *BRCA1* and nine in *BRCA2* [31]. None of the preliminary variants tested by Kluska et al. were present in our patient groups. In another study, Kowalik et al. revealed only the percentage of germline variants (pathogenic, variant of uncertain significance (VUS), and benign) detected in patients with breast and/or ovarian cancer (12.8%) and did not specify the percentage of germline variants in patients with breast cancer [33].

It has been shown that breast cancer with a *BRCA1* mutation is more often associated with medullary-like histopathology, TNBC and basal phenotype [34]. Our data and those of Kowalik [33] indicate that pathogenic *BRCA1/2* variants are more common in TNBC than in other types of breast cancer. We showed that pathogenic and likely pathogenic *BRCA1/2* variants detected in TNBC accounted for 44.4% of all cases in the studied population (4/9). In Świętokrzyskie Oncology Center in the south of Poland, 37% (7 out of 19) of germline pathogenic *BRCA1/2* variants detected in breast cancer were reported in TNBC patients [33]. The differences might be due to the group size and patient inclusion criteria, but our result of over 44% of pathogenic or likely pathogenic variants in TNBC indicates the importance of referring TNBC patients.

In the present study, we showed that the prevalence of pathogenic *BRCA1/2* variants in the TNBC group of patients was 26.7% (4/15). In an unselected Australian TNBC patient cohort, where 59% did not have any family history of breast or ovarian cancer, only 9.3% were found to have germline pathogenic *BRCA1/2* variants [32]. These results show the importance of the selection of enrolled patients.

Interestingly, in sample BR3/18, two pathogenic *BRCA2* variants were detected: germline NM_000059.3:c.5645C>A in exon 11 and somatic NM_000059.3:c.9097dupA in exon 23 (Table 2). Both mutations cause premature translation termination (stop codon); therefore, the somatic mutation will probably not affect treatment with PARP inhibitors. Nevertheless, the latest reports describe acquired, reversion mutations after platinum-based chemotherapy and PARPi therapy [35,36]. These somatic mutations (in the presence of a germline *BRCA* mutation) restore *BRCA1/2* function and are associated with a decrease in the response to current treatment and tumor progression [35,36]. The possibility of the detection of reversion mutations is one more argument for the superiority of *BRCA1/2* sequencing in tumor tissue. On the other hand, we also must admit that this single-nucleotide duplication was present in homopolymer region. Additional Sanger sequencing could be performed on tissue to better discriminate real somatic changes from a false-positive signal; however, 35% tumor content together with a homopolymer region makes this a difficult challenge.

In most of the examined patients with breast cancer (89.25%), we did not identify pathogenic *BRCA1/2* variants. In our experience, a recurrent germline mutation in *CHEK2* (c.444+1G>A, c.1100delC, del5395) and *PALB2* (c.509_510delGA and c.172_175delTTGT) can be found, respectively, in 1.98% and 1.98% of Polish patients with breast cancer using multiplex PCR, allele-specific PCR, (ASA-PCR), Restriction Fragment Length Polymomorphism (RFLP) and PCR-HRM (PCR-High Resolution Melting) (preliminary data). In 101 breast cancer patients, we have detected two mutations in *CHECK2* (c.444+1G>A (*n*=1) and del5395 (exon 10–11 deletion) (*n* = 1)) and two mutations in *PALB2* (c.509_510delGA (*n* = 1) and c.172_175delTTGT (*n* = 1)). All detected variants were confirmed by testing with other methods (Sanger sequencing) from the second independent blood sample withdrawal. Domagała et al., in their work, tested recurrent mutation *CHEK2* and *PALB2* recurrent mutation in patients with triple-negative and hereditary non-triple-negative breast cancer patients. Prevalence of germline variants was 3.5% (CHEK2 *n* = 7/202) and 0.5% (PALB2 *n* = 1/202), respectively [37]. In another study, the prevalence of *CHEK2* germline pathogenic variants (c.172_175delTTGTT; c.509_510delGA) in *BRCA1/2*-negative Polish patients with breast and ovarian cancer was 1.5% [38], and in another study, the prevalence of the c.509_510delGA mutation in *PALB2* in Polish breast and ovarian cancer patients was 0.6% (*n* = 4/648) [39]. Assessing the presence of pathogenic somatic variants in *CHEK2* and *PALB2* genes is not common practice. In breast cancer patients, somatic changes may be present in other genes involved in DNA repair: (1) homologous recombination (*ATM*, *ATR*, *CHEK1*, *CHEK2*, *BARD1*, *RAD51*, *NBS1*, *PALB2*, *FANCD2*), (2) non-homologous end joining (*DNA-PK*, *KU70/80*), (3) mismatch repair (*MLH1*, *MSH1*, *MSH6*, *PMS2*), (4) base excision repair (*APE1*, *XRCC1*, *ERCC2*) [40]. It is worth highlighting that there are different commercial panel tests to examine a wide range of genes related to breast cancer employing the NGS technique. A BreastNext multigene panel of 14 genes, excluding *BRCA1/2* (*ATM*, *BARD1*, *BRIP1*, *CDH1*, *CHEK2*, *MRE11A*, *MUTYH*, *NBN*, *PALB2*, *PTEN*, *RAD50*, *RAD51C*, *STK11* and *TP53*), was used to evaluate hereditary cancer predisposition in 874 patients tested [41]. However, only 7.4% patients with pathogenic or likely pathogenic variants were found using this panel.

In breast cancer patients, other molecular changes, such as epigenetic silencing of *BRCA1/2* and other genes, may be present. Esteller et al., in 2000, reported that hypermethylation and inactivation of *BRCA1* was detected in 13% of sporadic breast tumors [42]. Other potential changes are large insertions, deletions or structural rearrangement [43]. As mentioned earlier, LGR in the *BRCA1/2* genes are present in a small percentage of patients with breast and ovarian cancers, but the method used in the present study does not allow them to be detected. In general, LGR are not tested in the Polish population because of their low frequency (3.1–3.7%) [21,22].

Interestingly, in six samples, we detected VUSs. Previous studies have shown that co-segregation analysis and family history in a large cohort [44], as well as protein structure [45], in addition to the increasing number of functional studies, the development of computational prediction algorithms and database enlargement, allow VUSs to be reclassified as well [46,47].

The lack of unified reporting and VUS reclassification guidelines complicates comparisons between similarly designed studies within populations, in which the NGS technology was used, and encourages caution in extracting data from publications. For example, two Polish patient populations were examined at different oncology centers: Kowalik et al. [33] in 2018 considered pathogenic, VUS and benign variants using the ACMG recommendation, whereas Kluska et al. [31] in 2015 mainly used BIC, Condel Score and a literature search and did not mention VUS as a category separate from pathogenic and likely pathogenic variants in their study report. Depending on the biological material, variants detected can be classified using the ACMG guidelines (2015) [48] or TIER classification (2017) [49]. Another major difficulty concerning comparisons of studies is the different inclusion criteria for individuals selected for the study and the fact that some authors do not indicate whether the detected variants are germline or somatic [50].

In the present study, we identified one somatic variant in a tissue with 35% of tumor cells (Table 3), which is slightly lower but still in concordance with the data of other groups. Kowalik et al. reported that the lowest percentage of tumor cells with detectable pathogenic variants was 40% in ovarian cancer tissue samples [51], while Ellison et al. suggested that the starting material should contain at least 10% of tumor cells in order to detect low-frequency somatic variants [52].

NGS data analysis is the next key step. Laboratories use different programs and applications for secondary and tertiary analysis, depending on funds, experience and the number of tests performed. Various analysis algorithms, filters used in secondary analysis, strand biases, unbalanced strand mapping and variant calling can cause variability in the results of analyses [53,54]. Tools used in the analysis of sequencing data are crucial for the appropriate identification and interpretation of variants. It is important to choose the right tools and algorithms for secondary analysis. In our study, we observed differences in variant calling files (VCF) obtained from the same FASTQ files by means of different variant calling algorithms: for sample BR53/18 (Table 3). The accuracy (proportion of reads that are correctly mapped) and sensitivity (proportion of reads mapped to the reference genome) of tools used should be taken into account [55]. Tertiary analysis performed with different tools is more likely to have the same outcome if files from the secondary analysis of the same type are used.

## 5. Conclusions

In conclusion, our study has important value on a national scale owing to the well-selected population. We detected nine pathogenic variants in 8 out of 75 selected patients (10.7%), of which one was somatic and eight were germline variants. We can conclude the validation of NGS as an important bioinformatic procedure, the importance of screening both somatic and germline mutations and the importance of the role of additional susceptible genes in breast cancer. We present a preliminary step to estimate the size of the eligible population for PARPi treatment. Moreover, our study, alongside other similar ones, underlines the need to identify additional breast cancer susceptibility genes, particularly to explain the high percentage of hereditary cases. The next-generation sequencing technology offers new hope for this purpose.

## Figures and Tables

**Table 1:** Mann–Whitney U test for patients aged 25–45 (*n* = 46) and 46–69 (*n* = 29).

Group	Group Size	Sum of Ranks	U	σ	α_0.05_	Z
Patients aged 25–45	*n* = 46	1928.5	−303.5	89.83	1.96	10.47 > α
Patients aged 46–69	*n* = 29	921				

**Table 2:** Studied population of patients, genetically tested in 2003–2015 and negative for the presence of known mutations in the *BRCA1* gene (c.5266dupC, c.181T>G, c.4035delA, c.68_69delAG, c.3700_3704delGTAAA); diagnosed with breast cancer in 2003–2015; study group: *n* = 75 patients with breast cancer.

Clinical and Histopathological Features	Group Size	Number of Patients with Pathogenic Variants in *BRCA1/2*	Percentage of Patients with Pathogenic Variant
Age at diagnosis			
25–40	*n* = 29	*n* = 5	17.20%
41–45	*n* = 20	*n* = 2	10%
46–50	*n* = 14	*n* = 1	7.10%
51–69	*n* = 12	*n* = 0	0%
Tumor subtype (ER, PR and HER2 status)			
triple-negative	*n* = 15	*n* = 4	26.70%
ER+, PR-, HER2-	*n* = 8	*n* = 1	12.50%
ER-, PR-, HER2+	*n* = 4	*n* = 0	0%
ER+, PR+, HER2-	*n* = 42	*n* = 3	7.10%
other	*n* = 6	*n* = 0	0%
Content of tumor cells (%)			
5–10%	*n* = 5	*n* = 1	20%
11–20%	*n* = 9	*n* = 1	11.10%
21–40%	*n* = 36	*n* = 5	13.90%
41–60%	*n* = 13	*n* = 0	0%
61–100%	*n* = 12	*n* = 1	8.30%

**Table 3:** Analysis of 9 detected pathogenic variants in 75 selected patients with breast cancer.

						Classification					
						TruSeq Amplicon v.1.0.0 (Somatic Variant Caller)				Freebayes Variant Caller	
No	Sample Name	% of Tumor Cells	Gene	Change	VF (%)	Oncology Center Analysis			NGeneBio Classification	OncoKDM Belgium	Origin
						ACMG	TIER	EVA (EntroGen)			
1	BR 96/17	*30*	*BRCA1*	c.1687C>T p.Gln563Ter	79.0	Pathogenic PVS1 PM1 PM2 PP3 PP5	TIER I	Pathogenic	Pathogenic ENIGMA PVS1 PM2 PP5	Damaging c.1687C>T p.Q563 *	G
2	BR 14/17	*30*	*BRCA1*	c.4752C>G p.Tyr1584Ter	66.6	Pathogenic PVS1 PM1 PM2 PP3 PP5	TIER I	Pathogenic	Pathogenic ENIGMA PVS1 PM2 PP5	Damaging c.4689C>G p.Y1563 *	G
3	BR 7/18	*25*			72.3						G
4	BR 15/17	*20*	*BRCA1*	c.5186C>A p.Ala1729Glu	50.1	Pathogenic PP5 PM1 PM2 PP3	TIER I	Pathogenic	Pathogenic ENIGMA PS3 PM1 PM2 PP3 PP5	Damaging c.5123C>A p. A1708E	G
5	BR 10/18	*10*	*BRCA1*	c.5242A>T p.Lys1748Ter	47.0	Pathogenic PVS1 PM1 PM2 PP3 PP5	TIER I	Pathogenic	Pathogenic ENIGMA PVS1 PS3 PP5	Damaging c.5179A>T p.K1727 *	G
6	BR 11/17	*65*	*BRCA2*	c.7758G>A p.Trp2586Ter	65.9	Pathogenic PVS1 PM1 PM2 PP3 PP5	TIER I	Pathogenic	Pathogenic ENIGMA PVS1 PP5	Damaging c.7758G>A p.W2586 *	G
7	BR 53/18	*40*	*BRCA2*	c.8191C>T p.Gln2731Ter	74.9	Pathogenic PVS1 PM1 PM2 PP3 PP5	TIER I	Pathogenic	Pathogenic ENIGMA PVS1 PP5	Not detected	G
8	BR 3/18	*35*	*BRCA2*	c.9097dupA p.Thr3033Asn fsTer11	17.5	Pathogenic PVS1 PM1 PM2 PP3 PP5	TIER I	Pathogenic	Pathogenic ENIGMA PVS1 PM2 PP5	Not detected	S
9			*BRCA2*	c.5645C>A p.Ser1882Ter	47.4	Pathogenic PVS1 PM1 PM2 PP5	TIER I	Pathogenic	Pathogenic ENIGMA PVS1 PM2 PP5	Damaging c.5645C>A p.S1882 *	G

VF—variant frequency, G—germline, S—somatic. ACMG classification—Standards and guidelines for the interpretation of sequence variants of American College of Medical Genetics and Genomics and the Association for Molecular Pathology. Variants are classified into pathogenic, likely pathogenic, variants of uncertain significance, likely benign and benign. Classification according to TIER classification for somatic variants. Classify variants for 4 groups: TIER I—variants of strong clinical significance, TIER II—variants of potential clinical significance, TIER III- variants of unknown clinical significance, TIER IV—benign or likely benign variants. Ref. seq.: NM_007300.3 (*BRCA1*), NP_009231.2 (BRCA1), NM_000059.3 (*BRCA2*), NP_000050.2 (BRCA2).
